# Cognitive impairment after long COVID-19: current evidence and perspectives

**DOI:** 10.3389/fneur.2023.1239182

**Published:** 2023-07-31

**Authors:** Zhitao Li, Zhen Zhang, Zhuoya Zhang, Zhiyong Wang, Hao Li

**Affiliations:** ^1^Wangjing Hospital of China Academy of Chinese Medical Sciences, Beijing, China; ^2^The Affiliated Hospital of Shandong University of Traditional Chinese Medicine, Jinan, China; ^3^Xiyuan Hospital of China Academy of Chinese Medical Sciences, Beijing, China; ^4^Affiliated Hospital of Nanjing University of Chinese Medicine, Nanjing, China

**Keywords:** SARS-CoV-2, post-COVID cognitive impairment, neuroinflammation, mitochondrial dysfunction, neurodegeneration

## Abstract

COVID-19, caused by the SARS-CoV-2 virus, is a respiratory infectious disease. While most patients recover after treatment, there is growing evidence that COVID-19 may result in cognitive impairment. Recent studies reveal that some individuals experience cognitive deficits, such as diminished memory and attention, as well as sleep disturbances, suggesting that COVID-19 could have long-term effects on cognitive function. Research indicates that COVID-19 may contribute to cognitive decline by damaging crucial brain regions, including the hippocampus and anterior cingulate cortex. Additionally, studies have identified active neuroinflammation, mitochondrial dysfunction, and microglial activation in COVID-19 patients, implying that these factors may be potential mechanisms leading to cognitive impairment. Given these findings, the possibility of cognitive impairment following COVID-19 treatment warrants careful consideration. Large-scale follow-up studies are needed to investigate the impact of COVID-19 on cognitive function and offer evidence to support clinical treatment and rehabilitation practices. In-depth neuropathological and biological studies can elucidate precise mechanisms and provide a theoretical basis for prevention, treatment, and intervention research. Considering the risks of the long-term effects of COVID-19 and the possibility of reinfection, it is imperative to integrate basic and clinical research data to optimize the preservation of patients' cognitive function and quality of life. This integration will also offer valuable insights for responding to similar public health events in the future. This perspective article synthesizes clinical and basic evidence of cognitive impairment following COVID-19, discussing potential mechanisms and outlining future research directions.

## Introduction

COVID-19 is an acute infectious disease caused by the novel coronavirus (SARS-CoV-2) that can manifest as asymptomatic or severe pneumonia and multiple organ failure (1). Severe cases may present with high fever, dry cough, dyspnea, acute respiratory failure, septic shock, myocardial injury, and other complications (2). The global case fatality rate of COVID-19 is currently ~3.5%, but it can reach 15% or higher for high-risk groups, particularly older adults with underlying diseases (3, 4). Viral pneumonia, such as SARS and MERS, has been shown to cause cognitive impairment, especially in older individuals (5, 6). The mechanism may be related to direct viral infection of the CNS, extensive inflammatory response resulting from cytokine release and neurotoxicity, tissue hypoxia, and microangiopathy (7, 8). COVID-19 infection may also cause cognitive dysfunction. Some patients experience headaches, dizziness, and fatigue during the acute phase, and case reports have shown abnormal brain imaging findings (9, 10).

Patients recovering from COVID-19 have reported cognitive problems such as decreased memory and attention and sleep disorder. Studies have shown that some patients had abnormal results in neuropsychological tests, exhibiting declined working memory, language expression, and executive function (11–13). Given that the COVID-19 virus can invade the central nervous system by crossing the blood–brain barrier and nasal mucosa and infect neural cells, such as neurons, astrocytes, and microglia, that assist in their transport, it may directly damage the structure and function of the brain, leading to cognitive impairment (14, 15). In addition to the direct infection, COVID-19 may affect cognition through other mechanisms. First, the cytokine storm and widespread neuroinflammation triggered by the COVID-19 virus may disrupt neural circuits and connections, leading to neurotransmitter changes (16, 17). Second, sustained activation of the sympathetic nervous system and metabolic abnormalities can also damage the brain's vulnerable cognitive-related areas (18). Third, persistent hypoxemia caused by the disease can damage the brain tissue and affect neural activity (19). Finally, thrombus formation and microangiopathy caused by COVID-19 may also reduce cerebral blood flow, impairing cognitive function (20). Although most patients recovering from COVID-19 experience spontaneous recovery of cognitive function over time, some individuals, especially older adults and those with underlying diseases, may face long-term cognitive impairment (21, 22). Especially under the current situation of long COVID infections globally, it is necessary to strengthen the assessment of cognitive function in recovering individuals, paying particular attention to those with more severe conditions and longer hospital stays. Once abnormalities are detected, early identification and customized intervention should be provided. At the same time, accelerating the exploration of relevant mechanisms will help guide treatment and follow-up, thereby minimizing the impact of this complication and protecting the cognitive health of COVID-19 recoverers.

## Evidence of post-COVID cognitive impairment: neuropsychological and neuroimaging findings

After recovering from COVID-19, some patients have shown cognitive abnormalities, such as a decline in working memory, language expression, and executive function, as revealed by neuropsychological tests conducted after discharge. These findings suggest that COVID-19 may impair specific cognitive domains, such as executive control and working memory. In a survey of 969 people with SARS-CoV-2 infection 6–11 months ago, 26% of patients had mild cognitive impairment (23). A meta-analysis involving 2,049 people (24) suggested that COVID-19 patients had different MoCA scores than controls. COVID-19 recoverers showed lower general cognitive ability up to 7 months after infection. This was mainly reflected in visuospatial and executive function, while language and calculation abilities were relatively preserved (25). In executive function tests, some patients showed impaired executive control function, increased interference effects, and prolonged reaction times (26). This suggests that COVID-19 may damage the prefrontal cortex and executive control network, leading to attention-shifting and behavioral inhibition disorders. Baseler HA et al.'s study (27) found that in the working memory quiz, COVID-19 patients were significantly lower than non-COVID-19 patients. Some COVID-19 patients had retroactive interference disorders, making recalling and repeating the digit sequences they had just heard difficult. This suggests that COVID-19 may damage the hippocampus and related brain regions, affecting working memory's encoding and retrieval processes. In addition, a few studies have also found that COVID-19 patients have decreased language expressiveness and vocabulary. For example, in language fluency tests, the number of words generated by patients was lower than that in the healthy control group (28, 29). This may be due to the damage to brain regions closely related to language functions, leading to disorders of language fluency and vocabulary retrieval.

Brain imaging studies have found mild abnormalities in the brain structure and function of COVID-19 patients, possibly related to cognitive decline (30). Studies using structural magnetic resonance imaging (MRI) found that a few patients had mild atrophy of the hippocampus, gray matter, and mild ventriculomegaly (31). This suggests that COVID-19 may indirectly lead to brain tissue atrophy and ventriculomegaly by damaging neurons and synapses around the ventricles. These changes occur in brain regions with dense memory and cognitive networks, possibly related to decreased working memory and executive function. Positron emission tomography (PET) studies also found that glucose metabolism decreased in some patients' hippocampus, prefrontal cortex, and posterior cingulate cortex (32). This implies that these brain regions may have functional impairments after COVID-19 as glucose is the main energy source for these brain regions and is closely related to their activity. This could lead to decreased cognitive functions such as executive control, attention, and memory.

Another study compared the functional connectivity of the default network in 22 COVID-19 patients and healthy controls using resting-state fMRI. The results showed that the connectivity strength of the default network was weakened in the former group. The connectivity of the executive control network and emotional regulation network also changed (33). This suggests that COVID-19 can remodel brain functional networks and affect brain functional connectivity closely related to cognitive function.

In addition, relevant studies have also reported persistent cognitive impairments in COVID-19 patients. For example, a survey of 292 COVID-19 recoverers found that patients generally had physical damage, and these injuries were associated with more cognitive impairments (34). A 1-year prospective cohort study (35) investigated 1,438 COVID-19 survivors and 438 uninfected elderly individuals (all ≥60 years old) in China. By conducting cognitive tests at 6 and 12 months, respectively, changes in cognitive ability between the two groups were compared. The study excluded those with pre-existing cognitive impairment, a family history of dementia, or severe chronic diseases. Compared with the uninfected group, the cognitive decline rate in the infected group was significantly higher, especially in severe COVID-19 survivors. In these participants, the risk of mild cognitive decline was 4.87 times that of the uninfected group, and the risk of severe cognitive decline was 19 times that of the uninfected group. Non-severe COVID-19 was associated with a 1.71-fold increased risk (1.30–2.27) of early cognitive decline. Although the sample size was limited, these studies preliminarily show that a small proportion of COVID-19 patients may continue to suffer from cognitive impairments after recovery. Similarly, an Italian follow-up study of 50 recovered patients found that nearly one-fourth of patients had mild cognitive impairment 60 days after discharge, mainly manifested as decreased executive function and attention (12). A preliminary study of 100 patients by British scholars (36) also found that ~81% of COVID-19 patients who were not hospitalized had significant and persistent “brain fog” and fatigue symptoms after discharge, affecting their cognition and quality of life. This further confirms the risk of longer-term cognitive abnormalities in COVID-19 recoverers.

Multiple clinical study results support that memory and concentration may decrease persistently after COVID-19 infection. Some patients' neuropsychological tests and brain imaging performances suggest mild cognitive impairment. However, due to the limited sample size and lack of long-term follow-up in existing studies, we lack an in-depth understanding of the impact and incidence of this complication. Large-scale, long-term follow-up studies are needed to comprehensively assess changes in cognitive function after COVID-19 and provide evidence for early intervention.

## Potential mechanisms underlying post-COVID cognitive impairment

After recovery from COVID-19, some patients experience subjective memory loss, decreased concentration, and other cognitive problems. This may be related to the following mechanisms.

The COVID-19 virus can directly infect the central nervous system and damage the brain tissue and neurons, which may be an important mechanism leading to cognitive impairment. COVID-19 viral RNA and antigens have been detected in the cerebrospinal fluid and the brain tissue, indicating that the virus can cross the blood–brain barrier to invade the central nervous system (37). Viral surface proteins may promote the spread of protein aggregates in neurodegenerative diseases. The SARS-CoV-2 spike protein promotes the transmission of pathogenic seeds between cells *via* extracellular vesicles and direct intercellular transmission (38). The direct membrane contact mechanism spreads pathogenic seeds, and viral infection may accelerate the spread. Studies have found (39) that the spike protein is associated with memory loss after COVID-19. The SARS-CoV-2 spike protein activates the TLR4 receptor, causing neuroinflammation and microglial phagocytosis of synaptic proteins, leading to memory impairment. SARS-CoV-2-infected patients carrying TLR4-related gene polymorphisms are at higher risk of delayed memory impairment.

The COVID-19 virus can colonize the brain, leading to neuronal death and inflammation (7). This suggests that the virus can directly infect neurons, astrocytes, and vascular endothelial cells in the brain, damaging neural network connections and the integrity of the blood–brain barrier (40). Studies have found that the virus can aggregate around the hippocampus, brain stem, and cerebral blood vessels (41), the areas densely populated by neurons related to memory and cognition. Viral infection can damage synapses and the cytoskeleton, exacerbating neurodegenerative changes and loss of neural synapses (41). This may directly damage the neural circuits on which learning, memory, and executive control depend, leading to decreased cognitive function.

COVID-19 can induce cytokine storms and neuroinflammation, leading to neuronal damage and decreased cognitive function. Multiple inflammatory factors are significantly increased in COVID-19 patients, such as interleukin-6 (IL-6), tumor necrosis factor-α (TNF-α), and C-reactive protein (CRP) (42, 43). These factors can enhance microglial activity, promote neuroinflammation, and damage neurons and synaptic structures (44, 45). IL-6 can alter the balance of neurotransmitters, such as γ-aminobutyric acid in the brain, and damage neural connections and cognitive processes. TNF-α can damage the blood–brain barrier and enhance the entry of neurotoxic substances into the brain (46). This leads to loss of neural synapses, neuronal death, and reorganization of cognitive networks, resulting in decreased memory and executive function (47). Studies have found that neuroinflammatory markers significantly increase in brain regions closely related to cognitive function, such as the hippocampus, cerebellum, and anterior cingulate cortex (37). This indicates that neuroinflammation may have a greater impact on these brain regions and act with a viral infection to exacerbate neuronal damage and cognitive impairment. In addition, neuroinflammation is also associated with ventriculomegaly and decreased gray matter density, suggesting that it can lead to more extensive brain tissue damage and reorganization of cognitive networks. The cytokine storms and neuroinflammatory responses in COVID-19 patients can damage neurons, synapses, and cognitive network connections, which may be an important mechanism leading to their memory impairment and decreased executive function.

COVID-19 can lead to tissue hypoxia and microvascular lesions, affecting cerebral perfusion and the integrity of the blood–brain barrier, which may impair the function and cognition of brain areas such as the hippocampus. Ischemic lesions were detected in the brains of COVID-19 deceased patients. The S protein of the virus was detected in tissue sections, accompanied by tissue damage and cell death (39). COVID-19 patients often have hypoxemia and tissue hypoxia, and the oxygenated hemoglobin saturation of the brain tissue is also significantly reduced (48). This can lead to energy metabolism disorders and cell apoptosis in key brain areas such as the hippocampus, damaging neuronal structure and function (49). Studies have found that hippocampal volume and functional connectivity positively correlate with blood oxygen saturation (50). This suggests that hypoxia can directly damage the neural network of the hippocampus and affect cognitive processes. In addition, COVID-19 is often accompanied by microscopic vascular lesions, leading to cerebral vasculitis, thrombosis, and microhemorrhage (51). COVID-19 patients risk multifocal microvascular bleeding and ischemic lesions in the subcortical and deep white matter (52). This reduces cerebral perfusion, damages the blood–brain barrier, and increases neurotoxic substances entering the brain, aggravating neuronal damage (53). Animal experiments have also confirmed that hypoxia and cerebral microvascular lesions can act together to aggravate vascular endothelial damage and damage-related brain regions (54).

COVID-19 can lead to sympathetic excitation and metabolic abnormalities, affecting the brain environment and cognitive function. COVID-19 patients are often accompanied by sympathetic excitation and metabolic disorders such as hyperglycemia, hyperhomocysteinemia, and insulin resistance (55, 56). This can damage the brain's structure and function, such as the hippocampus, by affecting the energy supply and neurotransmitter balance in the key brain areas (57). Hyperglycemia can produce excess free radicals and oxidative stress, damage hippocampal neurons, and is associated with cognitive impairment (58). Hyperhomocysteinemia can also damage the long-term potentiation of the hippocampus, leading to decreased cognitive function (59). Insulin resistance can reduce the transport of apolipoprotein E and cholesterol, affect neurotransmitter synthesis and release, and is associated with cognitive decline (60). The sympathetic excitation and metabolic abnormalities caused by COVID-19, especially hyperglycemia, hyperhomocysteinemia, and insulin resistance, can damage hippocampal neurons, energy supply, and neurotransmitter balance, thereby affecting cognitive processes and functions.

**Figure 1:**
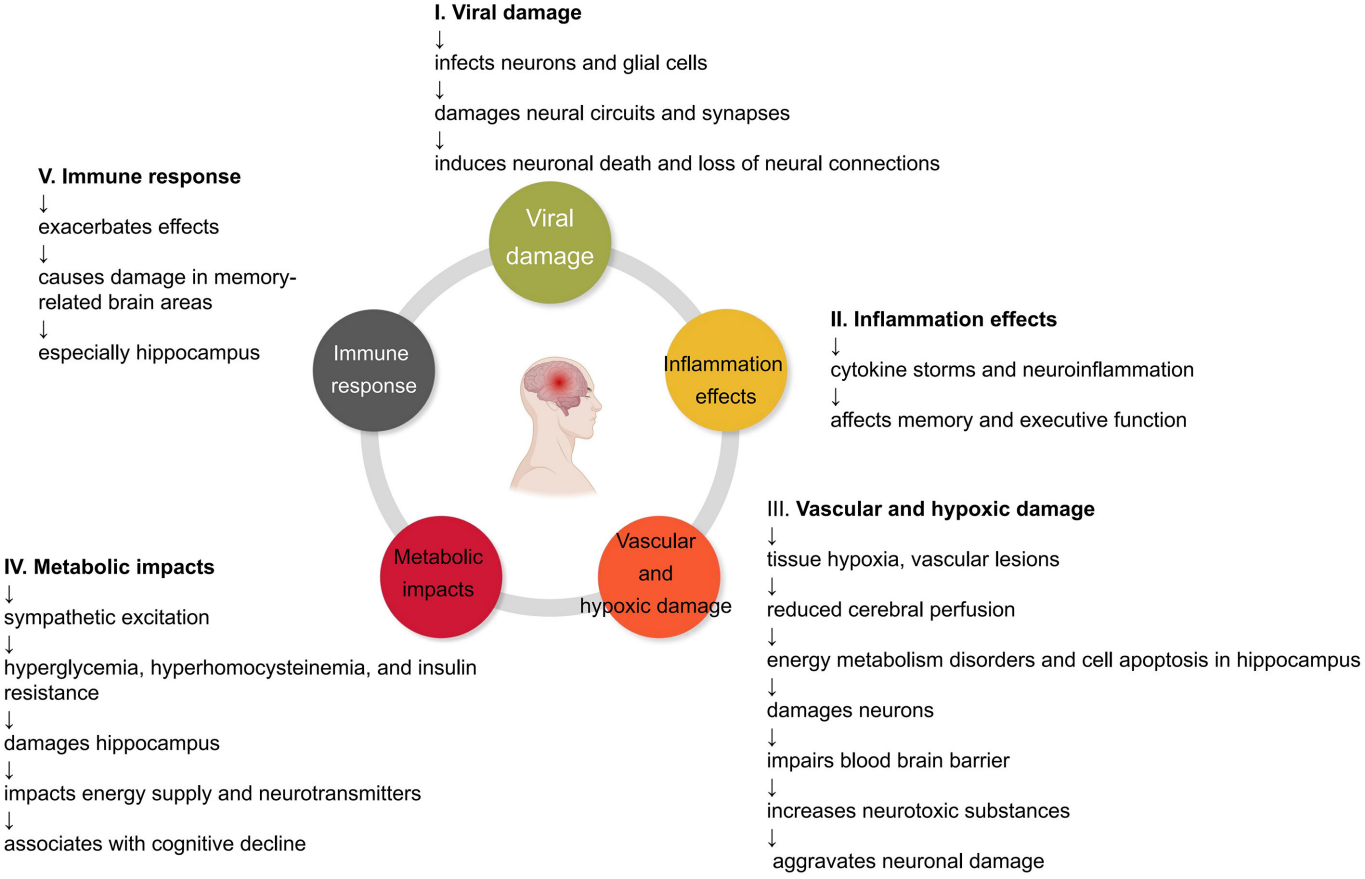
Impact of COVID-19 on the brain. Mechanisms by which COVID-19 may impair cognitive function and lead to memory loss. COVID-19 causes damage to the brain through direct viral infection, inflammation, vascular and hypoxic effects, metabolic impacts, and the body's immune response. These ultimately result in the loss of neural connections, reorganization of neural networks, and memory impairment.

As shown in Figure 1, COVID-19 may impair cognitive function and memory through multiple mechanisms. These various mechanisms should be considered comprehensively when assessing patients' cognitive status and developing intervention plans. Antiviral and immunosuppressive therapies may reduce damage from viral infections and inflammation; improving cerebral perfusion and oxygen supply can correct the effects of hypoperfusion and hypoxia; drug or training methods to regulate the sympathetic nervous system and metabolism are also expected to play a role. In summary, a deeper understanding of the relevant mechanisms will help guide the overall management and cognitive protection of COVID-19 recoverers.

## Further research on post-COVID cognitive impairment: key directions

Future research needs to explore the relationship between COVID-19 and cognitive impairment in more depth in the following aspects. First, COVID-19 patients still have some cognitive difficulties during recovery, including problems with memory, multitasking, processing speed, and attention. A large retrospective cohort study of over 2,30,000 patients found (61) that the risk of dementia within 6 months after COVID-19 infection was 2.33 times that of influenza patients during the same period. Studies using online cognitive tests and surveys to assess cognitive function found that 16% of patients developed new or worsening memory impairment, 18% had decreased attention, and 16% had decreased understanding, language expression, or other cognitive abilities. This suggests that COVID-19 may have long-term effects on the cognitive function of some patients, and we need to strengthen the monitoring and management of this population.

Second, studies have found that COVID-19 is associated with changes in the structure and function of the hippocampus and anterior cingulate cortex (62). The hippocampal volume of COVID-19 recoverers is smaller than that of the control group, suggesting neurodegenerative changes. In addition, COVID-19 is associated with weakened activation of the anterior cingulate cortex and ventricular enlargement, which can lead to decreased executive function. This suggests that COVID-19 may damage cognitive functions, such as learning, memory, and executive control, by affecting key brain regions such as the hippocampus and anterior cingulate cortex (63). COVID-19 can affect cognition through mechanisms (64–66) such as infecting neurons, activating microglia, and damaging vascular endothelial cells, while antiviral drugs may reduce neuronal damage caused by viral infection; immune regulation can alleviate viral-triggered neuroinflammation; and improving cerebral blood flow and oxygen supply can alleviate the effects of hypoxia and metabolic abnormalities on cognition. This provides important insights for clinical practice in cognitive rehabilitation and protection.

Studies have found a relationship between increased N-formylmethionine (fMet) levels and neutrophil activation in COVID-19 patients. Compared with healthy controls, COVID-19 patients, especially severe patients, have increased calprotectin, neutrophil extracellular traps (NETs), and fMet levels (67). Some studies show that inflammatory factors and cytokine levels are increased in the myelin and cerebrospinal fluid of Alzheimer's patients, suggesting that neutrophil activation may be involved in the pathogenesis of Alzheimer's disease (68). In addition, some studies have also found that inhibiting neutrophil function can alleviate cognitive impairment and pathological changes in Alzheimer's mice (69). This suggests that neuroinflammation and mitochondrial stress may play a role in COVID-19-induced neuronal damage. In addition, changes in the levels of proteins, such as Aβ42, tau, and neuron-specific enolase in the serum or cerebrospinal fluid, also provide clues to the mechanism of disease-induced neurodegeneration (70, 71). This may be related to the neurodegenerative diseases caused by the disease and provide a theoretical basis for biological markers and new drug development. Genomic studies can also help discover the molecular mechanisms by which the disease affects cognition. In addition, it has been suggested that certain natural compounds, including Ginkgo biloba extract, may hold promise in mitigating cognitive impairment resulting from long COVID-19 (72). However, these compounds' efficacy is yet to be firmly established, and further investigation is required to confirm their potential benefits.

## Conclusion

Although current research is not yet systematic and in-depth, COVID-19 recoverers seem to be at higher risk of cognitive impairment, which may be a key aspect of the potential long-term effects of the disease. Various mechanisms of COVID-19, such as viral infection, cytokine storm, microvascular lesions, hypoperfusion, hypoxia, and metabolic abnormalities, can act on the central nervous system together, causing neuronal dysfunction and damage, and then affecting learning, memory, and cognition (73). Recent studies have found that some COVID-19 recoverers have varying degrees of cognitive impairment, especially slower information processing speed, decreased working memory, and impaired executive function (36, 47). In addition, as a common symptom, post-COVID sleep disorder (PCSD) may negatively impact cognitive function. According to recent research (74), the incidence of PCSD may be over 70%, including various types of sleep disorders such as insomnia, hypersomnia, and daytime sleepiness (75, 76). Additionally, the severity of PCSD may be related to the severity of COVID-19 infection and treatment modalities. The COVID-19 virus may affect sleep through various mechanisms (74, 77, 78), including direct invasion of the central nervous system, induction of inflammatory response, and disruption of circadian rhythms. This suggests that we urgently need to conduct intensive monitoring and early identification for this population and develop targeted rehabilitation and intervention programs. Although the effects of COVID-19 on cognitive function are not fully understood, viral infection, neuroinflammation, and microvascular lesions may play an important role. This suggests that antiviral treatment, immune regulation, and improved microcirculation may benefit cognitive protection (79). Combined with multimodal neuroimaging, biomarker monitoring, and clinical assessment, this will help us stratify the risk of cognitive impairment and guide the individualized management of cognitive rehabilitation after COVID-19. In addition, as the scope of vaccination expands, we also need to pay attention to whether the vaccine can stimulate the immune response in the body and affect cognitive function to some extent. This will also be an important direction for future research (80). Combining basic and clinical research to comprehensively assess the impact of the COVID-19 epidemic on cognitive health will be critical.

## Data availability statement

The original contributions presented in the study are included in the article/supplementary material, further inquiries can be directed to the corresponding authors.

## Author contributions

HL and ZW contributed to the initial concept and perspectives. ZL, ZheZ, and ZhuZ developed the initial version of the manuscript. ZL, ZheZ, ZhuZ, and ZW reviewed and edited the manuscript along with HL. All authors read and approved the final manuscript.
